# Contrast-Enhanced Mammography and Deep Learning-Derived Malignancy Scoring in Breast Cancer Molecular Subtype Assessment

**DOI:** 10.3390/medicina62010115

**Published:** 2026-01-05

**Authors:** Antonia O. Ferenčaba, Dora Galić, Gordana Ivanac, Kristina Kralik, Martina Smolić, Justinija Steiner, Ivo Pedišić, Kristina Bojanic

**Affiliations:** 1 Department of Radiology, General Hospital Virovitica, 33000 Virovitica, Croatia; 2 Department of Translational Medicine, Faculty of Dental Medicine and Health Osijek, Josip Juraj Strossmayer University of Osijek, 31000 Osijek, Croatiamartina.smolic@fdmz.hr (M.S.); 3 School of Medicine, University of Zagreb, 10000 Zagreb, Croatia; gordana.ivanac@mef.hr; 4 Department of Diagnostic and Interventional Radiology, University Hospital Dubrava, 10000 Zagreb, Croatia; 5 Department of Medical Statistics and Medical Informatics, Faculty of Medicine Osijek, Josip Juraj Strossmayer University of Osijek, 31000 Osijek, Croatia; kristina.kralik@mefos.com; 6 Health Center of Osijek-Baranja County, 31000 Osijek, Croatia; justinija.pavkov@gmail.com; 7 Radiochirurgia Zagreb, 10431 Sveta Nedelja, Croatia; ivo.pedisic@gmail.com; 8 Department of Radiology, Health Center Osijek-Baranja County, 31000 Osijek, Croatia

**Keywords:** artificial intelligence, breast neoplasms, contrast media, deep learning, mammography

## Abstract

*Background and Objectives:* Contrast-enhanced mammography (CEM) provides both morphological and functional information and may reflect breast cancer biology similarly to Magnetic Resonance Imaging (MRI). *Materials and Methods:* This single-center retrospective study included 399 women with Breast Imaging Reporting and Data System (BI-RADS) category 0 screening mammograms who subsequently underwent CEM. A total of 76 malignant lesions (68 invasive cancers, 8 ductal carcinoma in situ (DCIS)) with complete imaging and pathology data were analyzed. Invasive cancers were classified into luminal A, luminal B, luminal B/Human Epidermal Growth Factor Receptor 2 (HER2)-positive, HER2-enriched, and triple-negative, and grouped as luminal (Group 1) versus HER2-positive/triple-negative (Group 2). *Results:* Luminal subtypes predominated (47 of 68, 69%), while 21 of 68 (31%) were HER2-positive or triple-negative. Most cancers appeared as masses with spiculated margins and heterogeneous enhancement. Significant differences were observed in mass shape (*p* = 0.03) and internal enhancement (*p* = 0.01). Luminal tumors were more often irregular and spiculated with heterogeneous enhancement, whereas the HER2-positive/triple-negative tumors more frequently appeared round with rim or homogeneous enhancement. Deep learning-derived malignancy scores (iCAD ProFound AI^®^) demonstrated good diagnostic performance (area under the curve (AUC) = 0.744, 95% confidence interval (CI) 0.654–0.821, *p* < 0.001). The median AI score was significantly higher in malignant compared with benign lesions (70% [interquartile range (IQR) 47–93] vs. 38% [IQR 25–61]; Mann–Whitney U test, *p* < 0.001). Among malignant lesions, iCAD scores varied across molecular subtypes, with higher median values observed in Group 1 versus Group 2 (87% vs. 55%), although the difference was not statistically significant (Mann–Whitney U test, *p* = 0.35). *Conclusions:* CEM features mirrored subtype-specific phenotypes previously described with MRI, supporting its role as a practical tool for enhanced tumor characterization. Although certain imaging and AI-derived parameters differed descriptively across subtypes, no statistically significant differences were observed. As deep-learning models continue to evolve, the integration of AI-enhanced CEM into clinical workflows holds strong potential to improve lesion characterization and risk stratification in personalized breast cancer diagnostics.

## 1. Introduction

Breast cancer (BC) remains the most common malignancy in women globally, with incidence still rising in many regions; if current trends persist, by 2050, BC is projected to reach 3.2 million new cases and 1.1 million deaths each year [1,2,3]. As a heterogeneous disease, its etiology is shaped by a multifactorial interaction of genetic susceptibility, hormonal and reproductive influences, lifestyle determinants, and environmental exposures, while advances in diagnostic modalities play a crucial role in facilitating early detection and improving clinical outcomes [4,5].

Imaging underpins the diagnostic pathway in BC [6]. Mammography remains the only modality proven to reduce mortality through early detection, yet it has limitations in sensitivity, especially in women with dense breasts [7,8]. To improve sensitivity, contrast-based imaging techniques have been developed. Breast Magnetic Resonance Imaging (MRI), with its excellent sensitivity and ability to depict tumor vascularity, has been used for high-risk screening and preoperative staging. More recently, contrast-enhanced mammography (CEM) has emerged as a clinically valuable alternative to MRI. Evidence from prospective studies and meta-analyses demonstrates that its diagnostic performance is comparable, though slightly inferior, to contrast-enhanced MRI in cancer detection [9,10,11]. Both modalities consistently achieve sensitivities exceeding 90%, even in dense breasts [9]. CEM confers practical advantages through greater accessibility, lower cost, lower false-positive rates, and improved tolerability in patient populations unable to undergo MRI, such as those with claustrophobia or MRI contraindications. As a result, CEM is increasingly used as a substitute for MRI when MRI is unavailable or contraindicated, and as a problem-solving tool in diagnostic workups [12,13].

Beyond early detection, precise characterization of BC is essential for guiding prognosis and treatment strategies [14]. Molecular subtype classification, typically determined by hormone receptor (estrogen [ER] and progesterone [PR]), Human Epidermal Growth Factor Receptor 2 (HER2), and Ki-67 status, has become a cornerstone of personalized oncology, as distinct subtypes (luminal A, luminal B, HER2-enriched, and triple-negative) are associated with different clinical outcomes and therapeutic responses [15,16].

There is growing evidence that imaging phenotypes correlate with these molecular subtypes. Prior studies using MRI and mammography have reported subtype-specific patterns: for example, triple-negative BCs often present as round or oval masses with circumscribed margins and rim enhancement, reflecting their high-grade, rapidly necrotizing nature [15,17]. In contrast, luminal subtype cancers (ER/PR-positive, usually lower grade) more frequently appear as spiculated masses with heterogeneous internal enhancement, and HER2-positive tumors are sometimes associated with pleomorphic microcalcifications due to accompanying Ductal Carcinoma In Situ (DCIS) [15,18]. These radiologic–pathologic correlations highlight the potential of advanced imaging techniques to noninvasively predict tumor subtype before surgery [17]. We hypothesize that CEM may be equally predictive of a tumor’s molecular and histopathologic characteristics.

Artificial intelligence (AI) has become an important adjunct in breast imaging, increasingly integrated into routine diagnostic workflows. Deep-learning approaches, particularly convolutional neural networks, have enabled the development of advanced algorithms capable of analyzing mammographic patterns with high diagnostic accuracy. These AI-based systems demonstrate performance comparable to experienced radiologists in cancer detection and may improve mammography interpretation by reducing false positives and enhancing lesion detectability, particularly in women with dense breast tissue [19,20]. Findings from a recent large cohort study similarly indicate that combining human reading with AI can increase breast-cancer detection compared with standard double reading, without performance differences across breast-density categories, supporting the complementary role of AI in population-based screening [21].

Recent radiomics studies have further highlighted the ability of CEM to reflect tumor biology. Zhu et al. [22] conducted a large multicenter radiomics analysis using CEM in molecular subtype prediction, achieving high discriminatory performance (AUC = 0.82 for luminal vs. non-luminal, 0.83 for HER2-enriched, and 0.69 for triple-negative cancers). These findings reinforce that CEM features can mirror molecular subtype-specific phenotypes and support its potential as a surrogate imaging biomarker of tumor biology.

While CEM shows promise in reflecting breast cancer biology, existing radiomics models are complex and not widely implemented clinically. Commercial AI systems, though effective for cancer detection, have not been fully explored for predicting molecular subtypes. This gap underscores the need to evaluate whether integrating readily available AI with CEM can noninvasively capture tumor heterogeneity, providing accessible, quantitative insights into molecular subtypes and supporting personalized treatment strategies.

In contrast to the complex radiomics models, our work examines whether a routinely used commercial AI system (iCAD ProFound AI) can reflect biological differences across molecular subtypes. The aim of the study was to examine the associations between CEM image features and BC molecular subtypes in a cohort of patients with suspicious mammographic findings. We evaluated CEM features of histopathologically confirmed cancers and analyzed their relationship with tumor subtype (grouped as luminal vs. HER2-enriched/triple-negative), alongside relevant clinical, imaging, and pathological variables (such as age, breast density, background parenchymal enhancement, and axillary nodal status). In addition, AI-derived malignancy probability scores obtained from the iCAD ProFound AI^®^ system were assessed to explore whether this commercially available algorithm provides complementary quantitative information related to tumor biology.

## 2. Materials and Methods

### 2.1. Study Design and Patient Selection

This single-institution retrospective observational study was conducted between October 2022 and December 2024 and was approved by the Ethics Committee (IRB number 03-2673-3/22). A cohort of 399 consecutive female patients presenting with abnormal screening mammography findings (Breast Imaging Reporting and Data System [BI-RADS] category 0) underwent CEM. Indications for CEM included suspicious shadows, distortions, asymmetry, or the accumulation of pathologic microcalcifications, which often necessitate further evaluation due to their indeterminate nature on initial mammograms. Clinical data, including age and family history of breast cancer (BC), were recorded for all patients. Eligibility was determined according to the following inclusion and exclusion criteria.

Inclusion criteria were:

- screening mammography abnormality categorized as BI-RADS 0,
- voluntarily agreed to participate in the study, and
- provided written informed consent.

Exclusion criteria were:

- contraindications to iodinated contrast administration (e.g., allergy or impaired renal function),
- history of prior breast cancer diagnosis or treatment.
- age under 40 years, and
- current pregnancy.

Inclusion criteria were selected to ensure a uniform cohort of women requiring further diagnostic evaluation after indeterminate screening findings, specifically BI-RADS 0 mammograms, for which CEM is clinically appropriate. Although the analysis was retrospective, patient inclusion was prospective: all patients were informed about the study at the time of their CEM examination and signed a written informed consent form for participation. Exclusion criteria were applied to avoid factors that could compromise patient safety or introduce bias. Contraindications to iodinated contrast material were excluded to ensure safe administration of CEM. Patients with a prior history of breast cancer were excluded because previous treatment can alter breast tissue appearance and enhancement patterns, potentially confounding imaging analysis. Women younger than 40 years of age and pregnant patients were excluded, as mammographic imaging is generally not recommended in these populations according to current clinical guidelines, and to avoid unnecessary radiation exposure. Together, these criteria ensured a consistent population suitable for evaluating CEM performance and its relationship to tumor biology.

Dual-energy CEM of both breasts was performed as part of the diagnostic workup, and biopsy was recommended for lesions showing suspicious enhancement on CEM. If the lesion was visible on ultrasound, a core needle biopsy was conducted under ultrasound guidance. For lesions not visualized sonographically, a vacuum-assisted biopsy (VAB) was conducted under stereotactic mammographic guidance, as contrast-guided biopsy was not available in our country at the time of the study. In a small subset of cases considered benign or cystic on ultrasound imaging, fine-needle aspiration was performed instead of core biopsy. In cases where CEM showed no suspicious enhancement corresponding to the mammographic lesion, the overall level of suspicion was downgraded, and patients were managed with imaging surveillance instead of immediate biopsy. Exceptions included morphologically highly suspicious microcalcifications, which were managed according to mammographic criteria regardless of post-contrast enhancement patterns.

**Figure 1:**
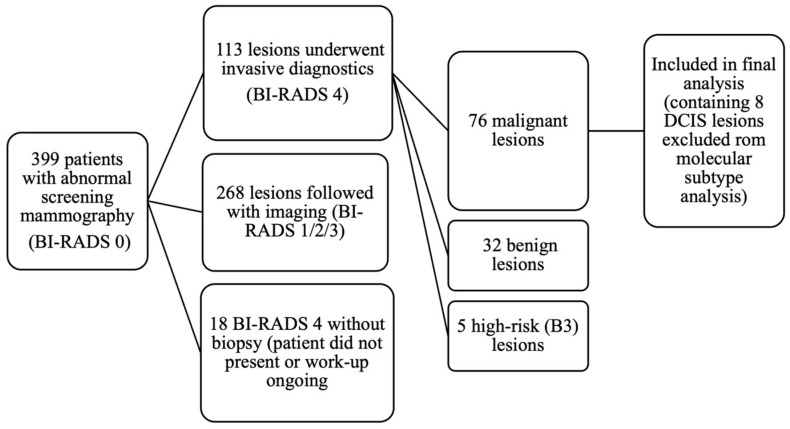
Flow diagram of patient selection and lesion inclusion in the study.

Out of 399 patients with abnormal screening mammography, invasive diagnostics were performed in 113 lesions (in 112 patients, as one patient had two lesions). These included 86 core needle biopsies, 14 fine-needle aspirations, and 13 vacuum-assisted biopsies. Pathology revealed 76 malignant lesions (invasive carcinoma or ductal carcinoma in situ [DCIS]), 32 benign lesions, and 5 high-risk (B3) lesions, which were managed according to current European guidelines (EUSOMA, EUSOBI, ESP-BWG, ESSO [23]). Among the malignant cases, 70 were diagnosed by core biopsy and 6 by VAB. In an additional 18 BI-RADS 4 cases, pathology results were not available in our institutional database, either because patients did not present for biopsy or their diagnostic workup was still ongoing at the time of data extraction. The patient selection process is summarized in Figure 1.

The remaining patients were managed conservatively with imaging follow-up. Specifically, BI-RADS 3 cases (*n* = 48) were scheduled for 6-month mammographic follow-up, whereas BI-RADS 1 (*n* = 34) and BI-RADS 2 (*n* = 175) underwent annual mammography; supplemental ultrasound was added in patients with dense breasts (ACR C and D). During follow-up (September 2022 to December 2024, with observation periods of up to 27 months), no cases of breast cancer have been detected among patients who did not undergo biopsy.

For this analysis, we focused on the 76 malignancies with complete imaging and histopathology data.

### 2.2. CEM Technique and Image Acquisition

All examinations were performed using a dual-energy full-field digital mammography system (Fujifilm Amulet Innovality, Tokyo, Japan). Patients were positioned in standard craniocaudal (CC) and mediolateral oblique (MLO) projections, and images of both breasts were acquired. An intravenous injection of the nonionic iodinated contrast medium (350 mg I/mL) was administered at a dose of 1.5 mL/kg body weight (maximum of 120 mL), followed by a 40 mL saline flush using a power injector through an antecubital vein. Imaging commenced approximately 2 min after contrast injection and was completed within 6–10 min to optimize visualization of contrast uptake while minimizing temporal washout.

Each breast was imaged in low- and high-energy modes during a single compression. Low-energy (low-dose, LD) images (26–31 kVp) were equivalent to standard digital mammography and provided anatomic information, while high-energy images (45–49 kVp) selectively captured the contrast signal. These were recombined by the manufacturer’s proprietary software to generate contrast-enhanced images highlighting areas of iodine uptake.

Quality control procedures were applied in accordance with institutional and manufacturer guidelines. All examinations were performed by certified mammography technologists following a standardized protocol to ensure reproducibility.

### 2.3. CEM Image Analysis

Lesion classification and descriptors were based on the Contrast-Enhanced Mammography (CEM) Lexicon [24], published as a supplement to the ACR BI-RADS^®^ Atlas in 2022, which adapts established mammography and breast MRI terminology for use in CEM and the ACR BI-RADS^®^ Atlas itself [25]. Two dedicated breast radiologists (15 and 5 years of experience, respectively) independently reviewed all images while blinded to histopathological results. Discrepancies were resolved in consensus during a joint reading session, thereby minimizing inter-observer variability.

Breast density was assessed on low-energy images according to the ACR BI-RADS^®^ classification: (A) almost entirely fatty, (B) scattered areas of fibroglandular density, (C) heterogeneously dense, which may obscure small masses, and (D) extremely dense, which lowers the sensitivity of mammography. Background parenchymal enhancement (BPE) was evaluated on the first post-contrast CC views and categorized as minimal, mild, moderate, or marked.

On CEM, lesions were categorized as either a mass or as non-mass enhancement (NME). Shape was categorized as oval, round, or irregular. Margins were classified as circumscribed or noncircumscribed, the latter including irregular and spiculated. Internal enhancement characteristics were described as homogeneous, heterogeneous, or rim enhancement. NME was described in terms of distribution and internal enhancement pattern. Distribution was categorized as focal, linear, segmental, regional, multiple regions, or diffuse. Internal enhancement patterns were classified as homogeneous, heterogeneous, or clumped.

Lesion conspicuity was assessed on both low-energy and recombined images and defined as low, moderate, and high conspicuity. The extent of lesion enhancement was categorized as partial, complete, beyond, or absent relative to the mammographic lesion.

Additional imaging features documented for each lesion included the presence of suspicious microcalcifications, defined as mammographic microcalcifications of a suspicious morphology and distribution. Axillary lymph node involvement was also documented. On CEM, lymph nodes were considered suspicious if new, significantly larger, or rounder compared to prior imaging; they were noted for clinical correlation and, when appropriate, recommended for further evaluation.

### 2.4. Histopathology and Molecular Subtype Determination

Tissue diagnosis was obtained through core needle biopsy, VAB, or needle aspiration, followed by surgical excision in malignant cases. Specialized breast pathologists performed histopathological evaluation according to standard protocols.

Each invasive breast carcinoma was classified histologically according to the WHO Classification of Tumors: Breast Tumors, 5th edition (2019) [26].

For clarification of diagnostic criteria and contemporary updates, additional authoritative review sources were consulted [27]. In addition, ductal carcinoma in situ (DCIS) was recorded separately. Immunohistochemistry was performed for ER, PR, HER2, and the Ki-67 proliferation index.

Molecular subtypes were defined as follows: Luminal A was ER and/or PR positive, HER2-negative, with low Ki-67, typically less than 20%. Luminal B was ER and/or PR positive, HER2-negative, with high Ki-67 (≥20%). If HER2 was positive, regardless of Ki-67 level, the tumor was classified as Luminal B HER2-positive. HER2-enriched (also called HER2-positive non-luminal) was defined as HER2 overexpressed/amplified and ER/PR negative. Triple-negative (basal-like) tumors were ER-, PR-, and HER2-negative [26].

**Figure 2:**
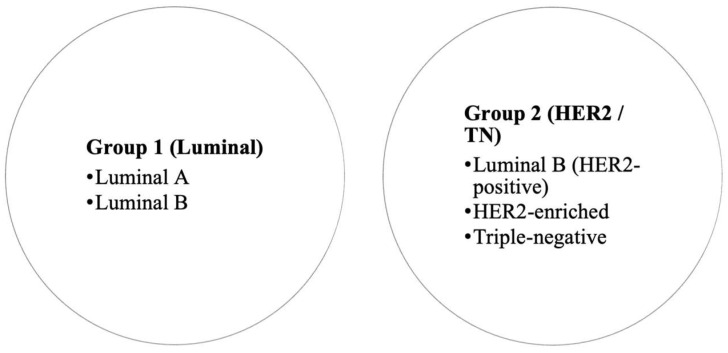
Grouping of breast cancer molecular subtypes for analysis.

For analysis, we consolidated subtypes into two broad groups based on receptor profile and expected tumor biology (Figure 2). Group 1 (Luminal) included luminal A and luminal B tumors, and Group 2 (HER2/TN) included luminal B/HER2-positive, HER2-enriched (non-luminal HER2-positive), and triple-negative cancers. This grouping reflects clinically more aggressive disease (Group 2) versus more indolent hormone-driven disease (Group 1). Since molecular profiling was not available for all DCIS cases in our institution, DCIS cases were not assigned a molecular subtype in the context of this grouping (they were analyzed separately in descriptive results but excluded from subtype-based comparative tests since “molecular subtype” typically refers to invasive carcinoma). Therefore, all comparative tests based on molecular subtype were limited to invasive carcinomas.

In addition to molecular subtype, the histological type of each cancer was recorded. The cohort included ductal carcinoma in situ (DCIS), invasive carcinoma of no special type (NST), invasive lobular carcinoma, papillary carcinoma, and mucinous carcinoma.

### 2.5. AI-Based Image Analysis

For all cases, malignancy probability was obtained from the commercial iCAD ProFound AI system (version 3.1.1.0; iCAD Inc., Nashua, NH, USA). We used the iCAD ProFound AI system, which was the only clinically implemented, commercially available AI tool in our institution, during the study period; it is also widely used in routine breast imaging practice. The ProFound AI score was computed exclusively on low-energy (LE) CEM images, which are equivalent to standard digital mammograms; recombined contrast images were not analyzed by the AI engine in this study. For each patient, the iCAD ProFound AI system generated Certainty of Finding Scores ranging from 0–100%, representing the estimated probability of malignancy for the detected lesion. The ProFound AI score is calculated at the lesion level, meaning that a single malignancy probability value is generated per lesion.

Scores were linked to histopathological outcomes (benign vs. malignant) and, for malignant cases, to the molecular subtype classification.

### 2.6. Statistical Analysis

Categorical variables were presented as absolute and relative frequencies, and differences between them were assessed using Fisher’s exact test. The normality of the distribution of continuous variables was evaluated with the Shapiro–Wilk test. Continuous data were expressed as median and interquartile range (IQR). The Mann–Whitney U test (with the Hodges–Lehmann estimate of the median difference and corresponding 95% confidence interval) was applied to compare medians between two groups, whereas the Kruskal–Wallis test was used for comparisons among three or more groups. Receiver operating characteristic (ROC) curve analysis was performed to evaluate the diagnostic performance of the iCAD score for predicting malignancy, including estimation of the area under the curve (AUC), sensitivity, specificity, and optimal threshold. All *p* values were two-tailed, and statistical significance was set at an α = 0.05. Statistical analyses were conducted using MedCalc^®^ Statistical Software version 23.3.7 (MedCalc Software Ltd., Ostend, Belgium; https://www.medcalc.org; 2025). The study report was prepared in accordance with the EQUATOR Network guidelines for reporting results in biomedical and health research.

## 3. Results

### 3.1. Patient and Tumor Characteristics

A total of 76 malignant lesions in 75 women form the basis of this analysis. The patients’ ages ranged from 43 to 87 years (median 62, IQR 56–68). Family history of breast cancer was reported in 9 (11.8%) patients. Of these patients, five reported a positive family history of BC in a first-degree relative, three in a second-degree relative, and one reported multiple affected family members, including both first- and second-degree relatives. The remaining 67 (88.2%) patients reported no family history.

Most women had intermediate breast density, with ACR category B in 39 (51%), A in 22 (29%), C in 13 (17%), and D in 2 (3%). Background parenchymal enhancement (BPE) was minimal in 59 (78%), mild in 13 (17%), and moderate or marked in 4 (5%) cases.

The majority of cancers were invasive carcinoma (68 of 76, 89.6%), while 8 (10.4%) were ductal carcinoma in situ (DCIS). Most invasive carcinomas were invasive carcinoma of no special type (NST), with less frequent occurrences of lobular, papillary, and mucinous histology (Table 1).

Among the 68 invasive cancers, luminal A and luminal B were the most frequent subtypes, together comprising 47 (69.1%) of cases, while luminal B/HER2-positive, HER2-enriched, and triple-negative subtypes (Group 2) accounted for 21 (30.9%) of cases. Thus, luminal cancers predominated in this cohort, while the more aggressive HER2-positive and triple-negative phenotypes were less common (Table 2).

### 3.2. CEM Lesion Presentation and Qualitative Imaging Features

All 68 invasive cancers were visible on the CEM recombined images. Of these, 55 (81%) lesions were identified as masses on CEM, while 13 (19%) lesions presented as non-mass enhancement (NME).

The majority of CEM-detected masses demonstrated spiculated margins, irregular shape, and heterogeneous internal enhancement as the predominant pattern (Table 3).

Non-mass enhancement (NME) lesions most often showed segmental or focal distribution, with heterogeneous enhancement being predominant (Table 4).

Suspicious microcalcifications on mammography were present in 35 of the 76 (46%) cancers, most frequently in luminal B/HER2-positive (8 of 14, 57%) and HER2-enriched (2 of 4, 50%) subtypes. Pathologically positive axillary lymph nodes were found in 28 of 68 invasive cancers (41.2%), while 40 (58.8%) cases showed no axillary nodal involvement.

### 3.3. Associations Between CEM Features and Molecular Subtypes

We examined the relationship between the qualitative imaging features described above and the molecular subtype groups (Group 1 vs. Group 2). For these analyses, the 8 DCIS lesions were excluded, as molecular profiling was not available for DCIS, and inclusion could have led to misclassification.

Background parenchymal enhancement (BPE) was most often minimal across all subtypes. In Luminal B, 22 of 24 (92%) cases demonstrated minimal BPE; in Luminal A, 15 of 23 (65%) had minimal BPE, with mild in 6 (26%), moderate in 1 (4%), and marked in 1 (4%). Luminal B/HER2-positive tumors showed minimal or mild BPE in 11 (79%) and 3 (21%) cases, respectively; HER2-enriched cancers demonstrated moderate enhancement in 1 (25%) case; Triple-negative cancers showed minimal in 2 (67%) and mild in 1 (33%). BPE did not differ significantly between groups (Fisher’s Exact test, *p* = 0.74). In Group 1, minimal BPE was present in 37 (79%), mild in 8 (17%), moderate in 1 (2%), and marked in 1 (2%); in Group 2, minimal in 15 (71%), mild in 5 (24%), moderate in 1 (5%), and none marked.

Breast density distribution varied across molecular subtypes, with density B predominating in luminal A (14 of 23, 61%) and luminal B (14 of 24, 58%). In luminal B/HER2-positive cancers, density A was more frequent (5 of 14, 36%), while HER2-enriched tumors showed higher rates of density C (2 of 4, 50%). Most triple-negative cases occurred in breasts with density A (2 of 3, 67%) and density C (1 of 3, 33%). Breast density did not differ significantly between the two groups (Fisher’s Exact test, *p* = 0.68). In Group 1, the majority of patients had density B (28 of 47, 60%), followed by densities A (9 of 47, 19%), C (9 of 47, 19%), and D (1 of 47, 2%), whereas in Group 2, densities A and B were equally represented (8 of 21, 38% each), with lower proportions of C (4 of 21, 19%) and D (1 of 21, 5%).

In Group 1, most masses were spiculated, whereas in Group 2, spiculated and irregular margins were equally common. Circumscribed margins were rare in both groups. Although a difference in distribution was observed, it did not reach statistical significance (Fisher’s Exact test, *p* = 0.24). Luminal A and B cancers were most often spiculated. Luminal B/HER2-positive tumors were equally irregular and spiculated. HER2-enriched tumors more frequently demonstrated circumscribed margins. Triple-negative masses were predominantly irregular.

Mass shape differed significantly between the groups (Fisher’s Exact Test, *p* = 0.03). In Group 1, most masses were irregular, whereas in Group 2, the majority were round. Oval shapes were equally distributed in both groups. Across molecular subtypes, luminal A and luminal B masses were most often irregular. In contrast, luminal B/HER2-positive and HER2-enriched tumors tended to be more frequently round. Triple-negative tumors showed no clear predominance of shape.

Mass internal enhancement patterns demonstrated a significant difference between the groups (Fisher’s Exact Test, *p* = 0.01). Group 1 lesions consistently exhibited heterogeneous enhancement, whereas Group 2 lesions displayed greater variability, including heterogeneous, homogeneous, and rim-enhancing patterns. Across molecular subtypes, luminal A and luminal B masses were uniformly heterogeneous. HER2-enriched tumors demonstrated an association with non-heterogeneous enhancement. Triple-negative tumors, with rim enhancement observed in approximately one-third of cases. Table 5 summarizes the mass characteristics on CEM.

Although NME was more frequent in Group 1 (11 of 47, 23%) compared to Group 2 (2 of 21, 10%), the difference was not statistically significant (Fisher’s Exact test, *p* = 0.42).

NME distribution did not differ significantly between the groups (Fisher’s Exact test, *p* = 0.42). In Group 1, segmental distribution was most common, followed by multiple regions and focal, while in Group 2, the two NME cases were regional and focal. Diffuse NME was rare, and linear distribution was not observed. Across molecular subtypes, luminal A and luminal B tumors demonstrated variable patterns, including segmental and focal distributions, whereas luminal B/HER2-positive and HER2-enriched tumors were infrequently associated with NME. Triple-negative cancers did not present with NME.

NME internal enhancement patterns were also not significantly different between the groups (Fisher’s Exact test, *p* > 0.99). In Group 1, heterogeneous enhancement predominated, with a subset showing clumped enhancement, whereas Group 2 cases were heterogeneous. Across molecular subtypes, luminal A and luminal B tumors displayed both heterogeneous and clumped patterns, while luminal B/HER2-positive and HER2-enriched tumors showed only heterogeneous enhancement. Triple-negative tumors were not associated with NME. Table 6 summarizes the NME characteristics on CEM.

The presence of suspicious microcalcifications did not differ significantly between the two groups (Fisher’s Exact test, *p* = 0.27). Microcalcifications were seen in 17 of 47 (36%) Group 1 cases and in 11 of 21 (52%) Group 2 cases. Suspicious microcalcifications were most frequent in luminal B/HER2-positive cancers (8 of 14, 57%).

Pathologically positive axillary lymph nodes were found in 6 of 23 (27%) luminal A, 11 of 24 (44%) luminal B, 8 of 14 (57%) luminal B/HER2-positive, 2 of 4 (50%) HER2-enriched, and 1 of 3 (33%) triple-negative cases. Grouping these, nodal involvement was 17 of 47 (36%) for Group 1 vs. 11 of 21 (52%) for Group 2 (Fisher’s Exact test, *p* = 0.29), without a significant difference. When analyzed by subtype, lymph node involvement tended to be more frequent in luminal B/HER2-positive cancers.

We did not observe a statistically significant difference in CEM lesion conspicuity (Fisher’s Exact test, *p* = 0.30). On LD images, similarly, no significant difference (82% high conspicuity in Group 2 vs. 58% in Group 1) was found (Fisher’s Exact test, *p* = 0.43). Lesion conspicuity was highest for luminal B, luminal B/HER2-positive, and triple-negative tumors, where the majority of lesions were highly conspicuous on both LD and CEM images. In contrast, luminal A tumors were less conspicuous on CEM. HER2-enriched cancers showed intermediate conspicuity. Table 7 summarizes lesion conspicuity across Groups.

We did not observe a statistically significant difference in the extent of enhancement between groups (Fisher’s Exact test, *p* = 0.47). The extent of enhancement was most often complete in luminal A (17 of 23, 74%) and luminal B (17 of 24, 72%) tumors, as well as in luminal B/HER2-positive (10 of 14, 71%) and triple-negative cancers (2 of 3, 67%). HER2-enriched tumors showed a more balanced distribution, with 2 of 4 (50%) complete and 2 of 4 (50%) beyond enhancement.

**Figure 3:**
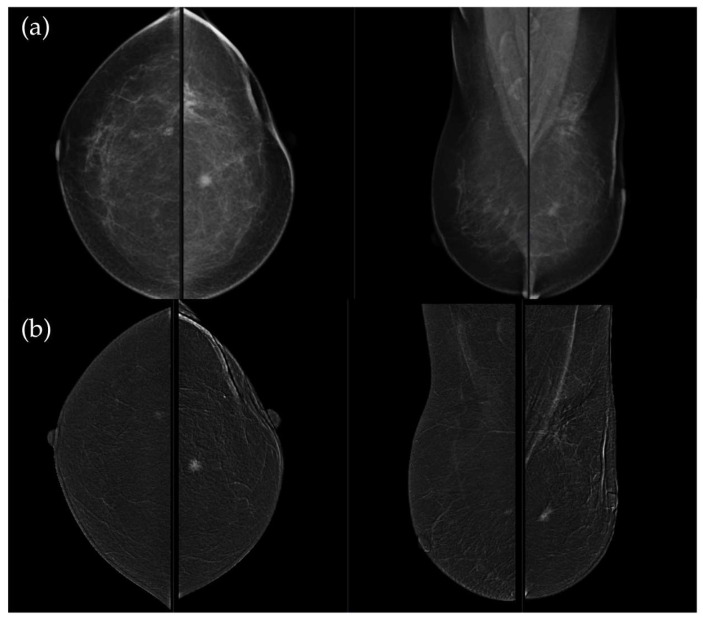
(**a**) Standard craniocaudal (CC) and mediolateral oblique (MLO) low-energy views reveal an irregular, spiculated mass of the left breast. The right breast shows a histopathologically confirmed fibroadenoma with dystrophic calcifications. (**b**) Recombined contrast-enhanced images (CC and MLO views) demonstrate early, complete heterogeneous enhancement. No pathologically enhancing axillary lymph nodes are detected. Histopathological analysis confirmed Luminal A breast carcinoma.

**Figure 4:**
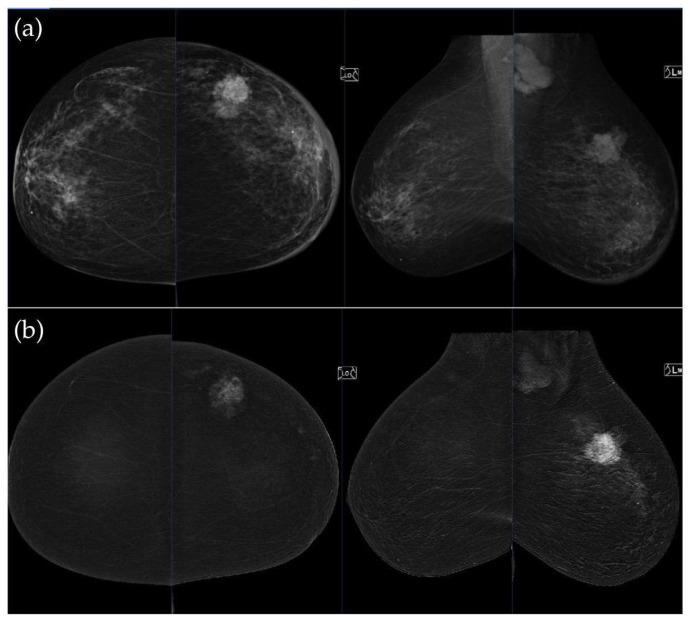
(**a**) Standard CC and MLO low-energy views reveal an irregular, spiculated mass with associated retraction of Cooper’s ligaments in the upper outer quadrant of the left breast. No suspicious microcalcifications are seen. The right breast appears unremarkable. (**b**) Recombined contrast-enhanced images (CC and MLO views) demonstrate an irregular, non-circumscribed lesion in the upper outer quadrant of the left breast with rapid early heterogeneous enhancement. Partially visualized axillary conglomerate on the left also demonstrates heterogeneous contrast enhancement in the early phase. Contralateral axilla is unremarkable. Histopathological analysis confirmed a Luminal B breast carcinoma.

**Figure 5:**
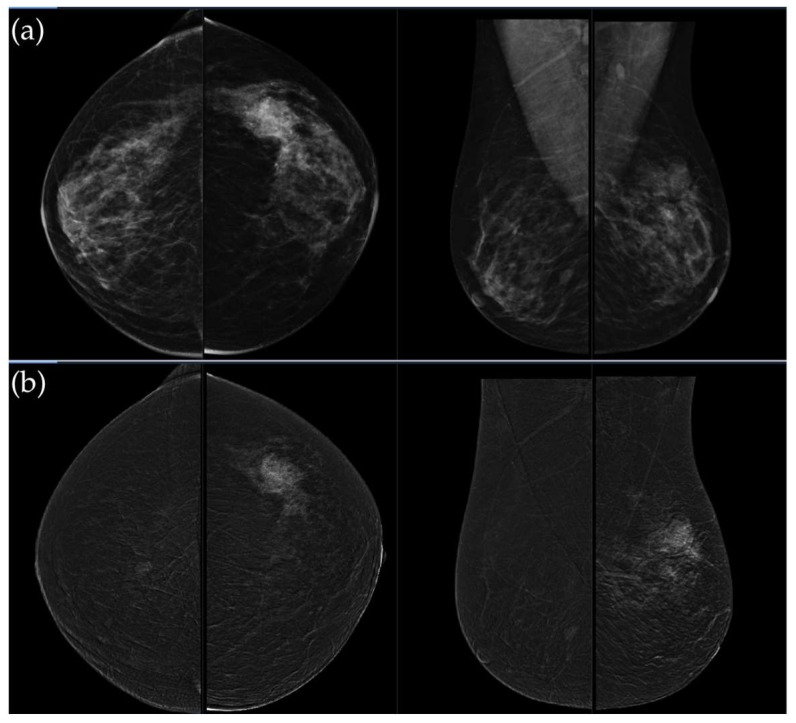
(**a**) Low-energy CC and MLO views reveal a focal asymmetry with associated suspicious microcalcifications and retraction of Cooper’s ligaments in the upper outer quadrant of the left breast. An oval, well-circumscribed mass with circumscribed margins is seen at the junction of the lower quadrants of the right breast. (**b**) Recombined contrast-enhanced images (CC and MLO views) demonstrate a large inhomogeneous enhancing area, with two closely situated, partially marginated masses in the upper outer quadrant of the left breast. The right breast lesion shows homogeneous early enhancement without washout on delayed images—findings suggestive of a benign lymph node or fibroadenoma. In the visualized portions of both axillae, no pathologically enhancing lymph nodes are identified. Histopathological analysis confirmed a HER2-enriched breast carcinoma.

**Figure 6:**
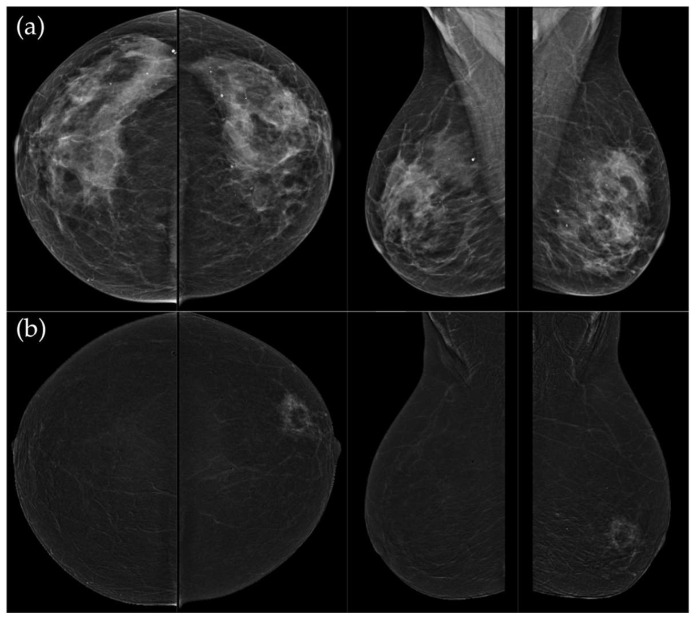
(**a**) Standard CC and MLO images reveal a relatively well-circumscribed, round to oval mass in the left retroareolar region, without suspicious calcifications. The right breast appears unremarkable. (**b**) On recombined contrast-enhanced images (CC and MLO), the same lesion demonstrates rim enhancement with irregular, heterogeneous internal contrast uptake. The enhancement shows early rapid contrast accumulation—a pattern that raises concern for malignancy. In the visualized portions of both axillae, no pathologically enhancing lymph nodes are identified. Histopathological examination confirmed the diagnosis of triple-negative breast carcinoma.

Figure 3, Figure 4, Figure 5 and Figure 6 present representative examples of CEM findings across molecular subtypes, with each case shown on both low-energy and recombined images.

### 3.4. Diagnostic Performance of iCAD AI Scoring

**Figure 7:**
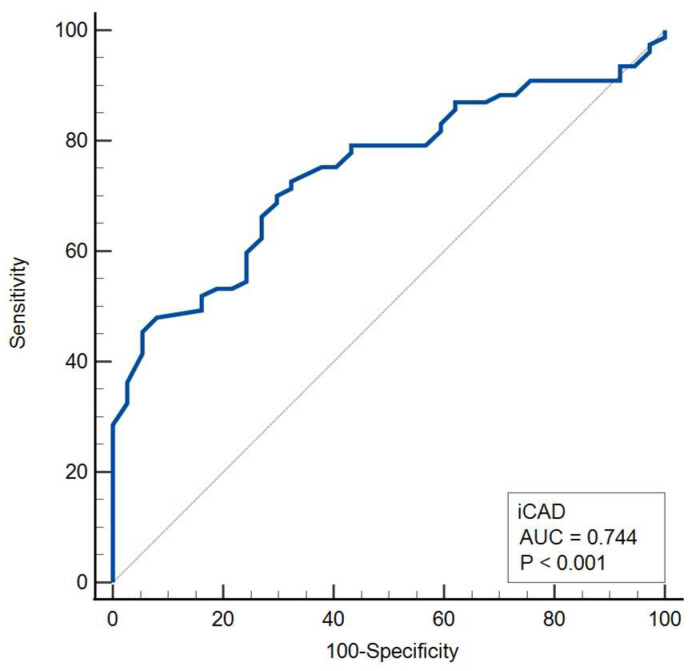
Receiver operating characteristic (ROC) curve for the iCAD score in malignancy discrimination.

The median iCAD malignancy probability was significantly higher in malignant compared with benign lesions (70% [IQR, 47–93%] vs. 38% [IQR, 25–61%]; median difference, 28% [95% CI, 17–40%]; Mann–Whitney U test, *p* < 0.001). Receiver operating characteristic (ROC) analysis demonstrated good discriminative ability of the iCAD score for malignancy (AUC = 0.744, *p* < 0.001), with an optimal cut-off value of 51%. The corresponding Youden index of 0.404 indicated a moderate overall diagnostic performance (Table 8, Figure 7).

**Figure 8:**
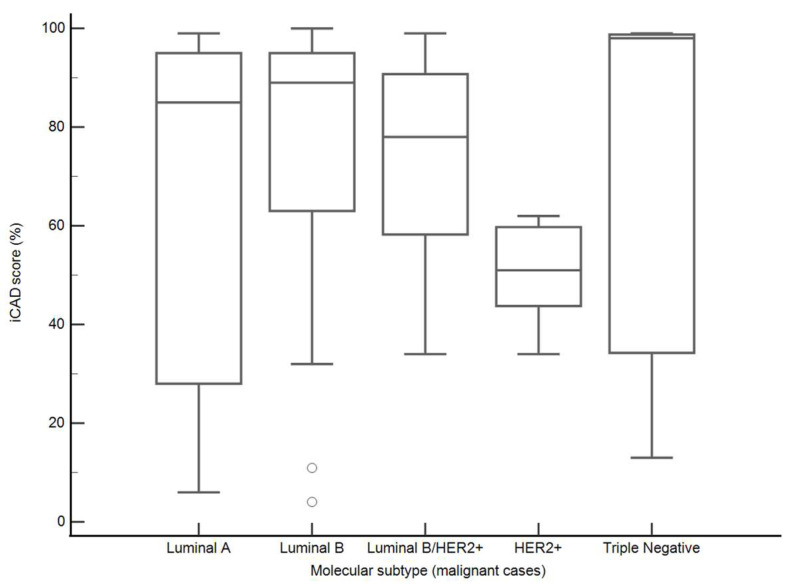
iCAD malignancy scores by molecular subtype (malignant cases). Boxplots represent medians (lines), interquartile ranges (boxes), and minimum–maximum values (whiskers).

Among malignant lesions with available molecular subtype classification (*n* = 68), iCAD scores varied across subtypes, with higher median values observed in triple-negative cancers (98% [IQR, 34–99%]), Luminal B cancers (89% [IQR, 63–95%]), and Luminal A cancers (85% [IQR, 28–95%]) compared with Luminal B/HER2+ (78% [IQR, 58–91%]) and HER2-positive (51% [44–60%]) tumors. However, these differences were not statistically significant (Kruskal–Wallis test, *p* = 0.40) (Figure 8).

When molecular subtypes were grouped as luminal (Group 1) versus HER2-positive/triple-negative (Group 2), the median iCAD scores were 87% (IQR, 63–91%) for Group 1 and 55% (IQR, 30–98%) for Group 2, with no statistically significant difference between the groups (median difference, −14.5%; 95% CI, −40 to 8; Mann–Whitney U test, *p* = 0.35).

**Figure 9:**
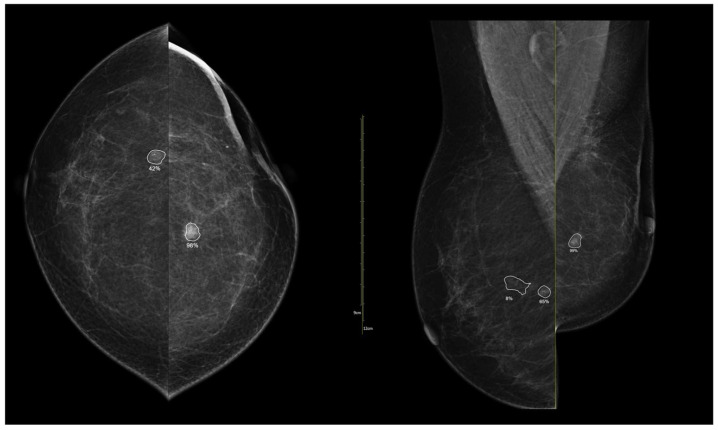
Example of iCAD ProFound AI^®^ output for a luminal A carcinoma. The lesion detected on the low-energy image is automatically outlined by the AI system with an assigned malignancy probability. The outline shows the regions of highest AI attention, corresponding to irregular shape and spiculated margins, typical of luminal tumors.

**Figure 10:**
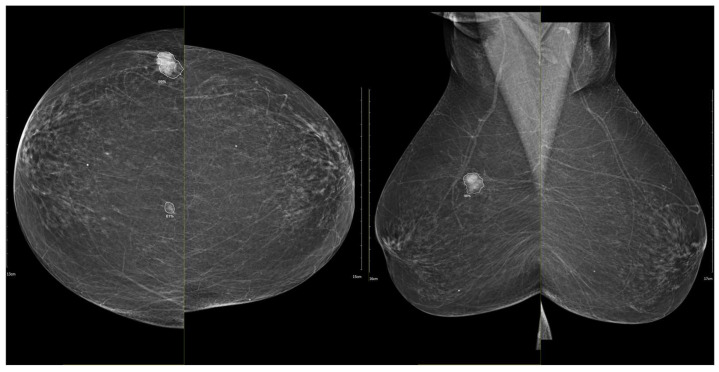
Example of iCAD ProFound AI^®^ output for a triple-negative breast carcinoma. The lesion detected on the low-energy image is automatically outlined by the AI system with an assigned malignancy probability. The outline shows the regions of highest AI attention, corresponding to oval shape and microlobulated, noncircumscribed margins.

To illustrate the behavior of the iCAD ProFound AI^®^ system across different biological phenotypes, Figure 9 and Figure 10 present representative examples corresponding to Group 1 (luminal) and Group 2 (HER2-positive/triple-negative) tumors. In both cases, the algorithm correctly identified the lesion with high confidence and localized regions corresponding to malignant radiomic traits. These findings should be interpreted in the context of case-level AI assessment rather than lesion-specific prediction.

## 4. Discussion

Conventional techniques, such as digital mammography, tomosynthesis, and breast ultrasound, remain widely used but are limited by reduced sensitivity, particularly in dense breasts. Contrast-enhanced mammography (CEM), by providing functional information through contrast enhancement, translates the principles of contrast-enhanced Magnetic Resonance Imaging (MRI) into a more accessible and cost-effective modality, improving lesion characterization and detection [28,29].

We investigated the relationship between CEM imaging features and breast cancer molecular subtypes in a cohort of 76 pathologically proven cancers. Our findings suggest that CEM may reveal imaging phenotypes that are associated with underlying tumor biology.

In our cohort, invasive carcinoma predominated, with invasive lobular, papillary, and mucinous carcinomas being less frequent. This mirrors large population and review data: invasive carcinoma accounts for ~40–80% [30]; invasive lobular ~15% [31]; mucinous ~4% [32]; and invasive papillary <3% [33] of cases. Thus, our cohort reflects the expected histologic spectrum, supporting generalizability. In our cohort, luminal A and luminal B together comprised 69% of invasive cancers, while HER2-positive and triple-negative tumors accounted for the remaining 31%. This distribution is comparable to a large population-based study from the United States, including 320,124 patients, in which Acheampong et al. [34] reported 72.6% luminal A, 11.2% luminal B, 4.8% HER2-enriched, and 11.3% triple-negative BCs.

Most women in our cohort had intermediate breast density and minimal background parenchymal enhancement, as typically observed in postmenopausal populations [35]. Most patients did not report a positive family history, which aligns with the fact that hereditary BC accounts for only 5–10% of all cases [36]. The median age of participants was 62 years, reflecting the age distribution commonly reported in population-based studies of BC patients [37].

Alongside these clinical and demographic features, we analyzed lesion morphology on CEM. Masses predominated, most frequently presenting with spiculated margins, irregular shape, and heterogeneous enhancement. Non-mass enhancements were less common and most often showed segmental or focal distribution with heterogeneous internal enhancement, consistent with the patterns described in the literature [38].

In our analysis, mass shape and internal enhancement appeared to be among the more distinctive CEM features across molecular subtypes, with luminal cancers more often irregular and spiculated with heterogeneous enhancement, and HER2-positive and triple-negative tumors more frequently presenting as round or oval with circumscribed margins and rim enhancement. Microcalcifications did not differ significantly across subtypes, although a possible trend toward higher frequency in HER2-positive cancers was observed, likely reflecting the frequent coexistence of a DCIS component. These findings are consistent with prior studies that have identified subtype-specific CEM patterns. Li et al. reported that luminal cancers, especially luminal B, were most often spiculated and calcified, HER2-enriched cancers commonly showed irregular morphology with malignant calcifications, while triple-negative cancers were significantly associated with round/oval shape, rim enhancement, and nodal involvement [39]. More recently, Cheng et al. confirmed the correlation of calcifications with HER2-positive tumors and rim enhancement with the triple-negative subtype [17]. Similarly, Piccolo et al. [40] explored the association between morpho-dynamic CEM features and prognostic factors, showing that specific morphological and enhancement characteristics, such as irregular shape, spiculated margins, and enhancement kinetics, were significantly correlated with biological aggressiveness indicators, including HER2, PR, and Ki-67. Although their analysis did not directly address molecular subtypes, their results reinforce the concept that CEM morphological traits may mirror tumor biology, complementing our findings and supporting the use of CEM as a potential imaging biomarker of breast cancer phenotype. Likewise, Marzogi et al. [41] investigated whether the level of contrast enhancement on CEM correlates with the presence and biological aggressiveness of breast cancer. They demonstrated that invasive and high-grade tumors typically exhibited strong or distinct enhancement, whereas in situ and luminal lesions more often showed subtle or absent enhancement. Moreover, enhancement intensity was positively correlated with Ki-67 expression and inversely related to hormone receptor positivity, suggesting that CEM enhancement strength reflects tumor proliferation and aggressiveness. These results further support the growing evidence that enhancement characteristics on CEM are linked to the underlying biological behavior of breast cancers.

Although obtained using MRI rather than CEM, Bartsch et al. [42] demonstrated that imaging-derived physiological parameters such as tumor oxygenation and perfusion can reflect molecular subtype–specific biology, reinforcing the link between imaging phenotype and tumor microenvironment.

Prior breast MRI studies established some hallmark imaging differences among subtypes. For example, triple-negative cancers are known to present as well-circumscribed masses, often with rapid contrast uptake and central necrosis leading to rim enhancement on MRI [15,43]. Consistent with this, Moffa et al. [44] reported that 58% of triple-negative cancers demonstrated circumscribed margins, 75% showed rim enhancement, and 42% exhibited intralesional necrosis, all of which were significantly associated with the subtype. Luminal A/B cancers, being lower grade, more often present as spiculated masses without rim enhancement [15,45]. Our CEM-based observations support these MRI findings.

Axillary lymph node metastases were present in 41%. Axillary lymph node involvement showed a non-significant tendency toward being more common in more aggressive subtypes (Luminal B/HER2-positive, HER2-enriched), though not statistically significant in our sample. Previous studies have described higher nodal positivity in aggressive subtypes such as HER2-positive and triple-negative cancers. Pereira et al. [46] reported that ER-negative clusters, including HER2-positive and triple-negative cancers, were significantly associated with increased rates of axillary metastases compared with ER-positive tumors, specifically Luminal A and Luminal B subtypes. The absence of significant differences in our study is most likely attributable to the limited number of HER2-enriched and triple-negative cases. In addition, our study assessed axillary lymph nodes on CEM, which demonstrated suspicious features in selected cases. Consistent with the findings of Lobbes et al. [47], CEM may be combined with targeted ultrasound to provide comprehensive locoregional staging in a single session, potentially reducing the need for additional MRI. Although CEM has a limited field of view and ultrasound is generally adequate for evaluating axillary and supraclavicular lymph nodes, assessing internal mammary lymph nodes still represents a challenge and limits the ability to achieve fully comprehensive nodal staging.

On lesion conspicuity, luminal A lesions in our data were significantly less conspicuous on CEM; luminal B, luminal B/HER2+, and triple-negative cancers were more clearly visible. These findings indicate that BCs across molecular subtypes were adequately visualized on CEM, supporting its robustness as a detection tool regardless of tumor subtype. However, other groups have reported different results: Bellini et al. [48] observed that lesion conspicuity was significantly higher in HER2-positive and triple-negative cancers compared to Luminal A tumors, suggesting that conspicuity may partly reflect tumor aggressiveness. Differences between studies may be explained by cohort size, subtype distribution, and methodological factors.

In our cohort, the extent of enhancement was most often complete across all molecular subtypes, with no significant group differences. Recent work suggests that quantitative metrics such as contrast-to-noise ratio may better distinguish malignant from benign lesions than subjective assessment alone [49]. Our findings, therefore, indicate that categorical evaluation of the extent of enhancement may have limited discriminatory value for molecular subtyping, and future studies should explore quantitative approaches.

On breast MRI, higher levels of BPE have been associated with premenopausal status, hormonal stimulation, and an increased risk of breast cancer [50]. In our study, most patients demonstrated minimal or mild background parenchymal enhancement (BPE), with some variability across molecular subtypes. However, due to the limited sample size, especially in HER2-positive and triple-negative groups, these findings should be interpreted with caution. We did not perform a direct comparison with MRI, which is an important limitation. Ferrara et al. [51], in a much larger cohort of 343 patients, found only fair agreement between CEM and MRI in BPE assessment, with postmenopausal women consistently showing lower levels. Our results, showing predominantly low BPE in postmenopausal women, are consistent with these observations. These results highlight the need for larger studies to validate the clinical significance of CEM-derived BPE and its relationship to tumor biology.

From a clinical perspective, subtype-specific CEM features may support preoperative planning. Findings such as rim enhancement, round mass shape, or suspicious microcalcifications can indicate more aggressive subtypes, prompting earlier biopsy or neoadjuvant therapy. In this regard, Vidali et al. [52] demonstrated that CEM can also effectively monitor response to neoadjuvant chemotherapy across molecular subtypes. In their two-center study of 174 patients, CEM achieved an overall sensitivity of 66% and specificity of 75% in predicting pathological complete response, with the highest sensitivity observed in triple-negative and HER2-positive tumors. The authors found that CEM performance varied according to tumor biology, with more aggressive subtypes exhibiting higher enhancement reduction after therapy. These findings highlight the potential role of CEM not only in subtype characterization but also in assessing treatment response and supporting individualized management strategies. In centers without an MRI, CEM provides a practical alternative for triage and treatment planning.

Previous evaluations of iCAD systems in mammography have primarily focused on detection performance. Ko et al. [53] demonstrated that the iCAD MammoReader increased cancer detection by 4.7% with a sensitivity of 94%. Cole et al. [54] and Cascio et al. [55] confirmed the consistent diagnostic performance and high specificity of iCAD in digital mammography. However, these studies assessed CAD as a detection aid, without exploring the biological meaning of its output. In contrast, our analysis extends the use of iCAD to biological interpretation, showing that the AI-derived malignancy probability score not only differentiates malignant from benign lesions but also tends to be higher in more aggressive, non-luminal subtypes; however, this trend did not reach statistical significance and should therefore be interpreted as exploratory rather than confirmatory. These findings suggest that commercial AI scoring systems may encode imaging biomarkers that reflect tumor phenotype and aggressiveness, thereby bridging diagnostic detection and biological insight, warranting validation in larger studies.

This study has several limitations. First, molecular profiling was not available for cases of ductal carcinoma in situ (DCIS), which were therefore excluded from subtype-based comparisons to avoid misclassification; these lesions were described only in the general results. Second, the overall sample size was modest, and Group 2 subtypes were underrepresented, including Luminal B/HER2-positive (*n* = 14), HER2-enriched (*n* = 4), and triple-negative cancers (*n* = 3). No a priori power analysis was performed, and the study was therefore not powered to detect small differences between subgroups. While a clear pattern was observed in these cases, the low numbers limit statistical power, and larger cohorts would be needed to better quantify the prevalence of features such as rim enhancement. Accordingly, findings related to Group 2 (HER2-positive and triple-negative cancers) should be interpreted with caution and considered exploratory in nature; these subgroup analyses are best regarded as hypothesis-generating rather than definitive. Third, the study was conducted at a single institution, which may introduce selection bias related to patient population and imaging protocols. Fourth, lesion assessment relied primarily on qualitative descriptors; although standardized criteria were applied, these remain subject to inter-reader variability. We attempted to mitigate this by employing two experienced radiologists who reached consensus on all cases. Inter-observer agreement statistics were not calculated, as quantifying reader variability was not an aim of this study. Fifth, while the iCAD malignancy score was analyzed as an AI-derived quantitative parameter, other radiomic or deep-learning features were not incorporated, which could provide additional objective and reproducible descriptors. Sixth, although multiple statistical comparisons were performed, no formal correction for multiple testing (e.g., Bonferroni) was applied, as this approach may be too conservative in predefined, exploratory analyses. However, the possibility of a type I error cannot be excluded and should be considered when interpreting the results. Finally, no direct comparison with breast MRI was performed, which would have allowed a more comprehensive evaluation of the relative diagnostic and predictive performance of CEM. These limitations suggest caution in generalizing our findings but also point toward clear directions for future research.

Future research should validate these findings in larger, multicenter cohorts with balanced representation of all molecular subtypes, with particular emphasis on the underrepresented HER2-enriched and triple-negative cancers. In addition to descriptive correlations, future work should incorporate quantitative approaches, including radiomics and machine learning, which have already demonstrated promising results in improving the accuracy of molecular subtype prediction [7,56,57]. In our work, the integration of a commercial AI scoring system (iCAD ProFound AI system) represented a preliminary step toward this goal, showing the potential of combining clinically available AI tools with CEM to explore biological correlations. In line with this, Mota et al. [58] recently introduced a deep-learning approach based on standard mammography for direct molecular subtype prediction. Using a large dataset of 1397 images and a ResNet-101 model, their framework achieved promising accuracy, particularly for HER2-positive and triple-negative cancers. This study highlights the feasibility of integrating artificial intelligence with mammographic imaging to noninvasively predict tumor biology, potentially reducing the need for repeated biopsies. Such AI-driven methods could be further extended to CEM datasets, offering an opportunity to combine morphological, enhancement, and computational biomarkers for more precise, image-based molecular classification. Our approach differs in that it evaluates a commercially implemented AI tool rather than a custom deep-learning model, highlighting its potential for practical implementation in routine clinical settings and its capacity to facilitate future clinical translation. While the iCAD ProFound AI^®^ system has been extensively validated for breast cancer detection in screening mammography, its application beyond the original intended use, such as molecular subtype characterization, has been less explored. Therefore, the present analysis should be regarded as exploratory, and further validation using independent datasets will be valuable to confirm the generalizability of these findings.

Moreover, direct head-to-head comparisons with breast MRI in the same patient population would provide a more comprehensive assessment of the relative diagnostic and predictive performance of CEM.

## 5. Conclusions

Our study supports the growing evidence that contrast-enhanced mammography (CEM) is not only a sensitive diagnostic tool but may also reflect phenotypic information potentially related to tumor biology. Specific CEM features, such as mass shape and enhancement pattern, were associated with breast cancer subtypes. Furthermore, integration of the iCAD ProFound AI system suggests that quantitative AI scores showed exploratory trends across subtypes, although these were not statistically significant and should be interpreted with caution. With its practicality and expanding availability, CEM enhanced by AI may hold promise as a valuable tool in precision breast cancer diagnostics, pending confirmation in larger studies.

## Figures and Tables

**Table 1:** Histological types of malignant lesions (*n* = 76).

Histological Type	*n*	%
**Invasive carcinoma of no special type (NST)**	57	75.0
**Invasive lobular carcinoma**	6	7.9
**Ductal carcinoma in situ (DCIS)**	8	10.5
**Papillary carcinoma**	3	3.9
**Mucinous carcinoma**	2	2.6

**Table 2:** Molecular subtypes of invasive breast cancers (*n* = 68).

Group	Subtype	*n*	%
**Group 1**	Luminal A	23	33.8
	Luminal B	24	35.3
**Subtotal Group 1**		47	69.1
**Group 2**	Luminal B/HER2-positive	14	20.6
	HER2-enriched	4	5.9
	Triple-negative	3	4.4
**Subtotal Group 2**		21	30.9

**Table 3:** Morphological and enhancement characteristics of CEM-detected masses (*n* = 57).

Feature	Category	*n*	%
**Margins**	Spiculated	36	63.2
	Irregular	19	33.3
	Circumscribed	2	3.5
**Shape**	Irregular	24	42.1
	Round	21	36.8
	Oval	12	21.1
**Internal Enhancement**	Heterogeneous	52	91.2
	Rim enhancement	2	3.6
	Homogeneous	2	3.6

**Table 4:** Distribution and internal enhancement of non-mass enhancement (NME) lesions (*n* = 20).

Feature	Category	*n*	%
**Distribution**	Segmental	7	35
	Focal	6	30
	Multiple regions	3	15
	Regional	2	10
	Diffuse	1	5
	Linear	1	5
**Internal Enhancement**	Heterogeneous	15	75
	Clumped	5	25
	Homogeneous	0	0

**Table 5:** Comparison of mass characteristics (shape, margins, and internal enhancement) between Group 1 and Group 2 invasive cancers on CEM.

Feature	Category	Group 1 (*n* = 36)	Group 2 (*n* = 19)	*p* Value *
**Shape**	Irregular	19 (53%)	4 (21%)	**0.03**
	Round	9 (25%)	11 (58%)	
	Oval	8 (22%)	4 (21%)	
**Margins**	Spiculated	25 (69%)	9 (47%)	0.24
	Irregular	10 (28%)	9 (47%)	
	Circumscribed	1 (3%)	1 (5%)	
**Internal enhancement**	Heterogeneous	36 (100%)	15 (79%)	**0.01**
	Homogeneous	0 (0%)	2 (11%)	
	Rim enhancement	0 (0%)	2 (11%)	

* Fisher’s Exact Test. Statistically significant values are denoted in bold.

**Table 6:** Comparison of NME characteristics (distribution and internal enhancement patterns) between Group 1 and Group 2 invasive cancers on CEM.

Feature	Category	Group 1 (*n* = 11)	Group 2 (*n* = 2)	*p* Value *
**Distribution**	Segmental	5 (45%)	0 (0%)	0.42
	Multiple regions	2 (18%)	0 (0%)	
	Focal	2 (18%)	1 (50%)	
	Diffuse	1 (9%)	0 (0%)	
	Regional	1 (9%)	1 (50%)	
	Linear	0 (0%)	0 (0%)	
**Internal Enhancement**	Heterogeneous	7 (64%)	2 (100%)	>0.99
	Clumped	4 (36%)	0 (0%)	
	Homogeneous	0 (0%)	0 (0%)	

* Fisher’s Exact Test.

**Table 7:** Lesion conspicuity on LD and CEM by Group.

Modality	Category	Group 1 (*n* = 47)	Group 2 (*n* = 21)	*p* Value *
**LD**	Low	6 (13%)	2 (10%)	0.43
	Moderate	13 (28%)	3 (14%)	
	High	28 (60%)	16 (76%)	
**CEM**	Low	7 (15%)	2 (10%)	0.30
	Moderate	14 (30%)	3 (14%)	
	High	26 (55%)	16 (76%)	

* Fisher’s Exact Test.

**Table 8:** ROC analysis of the iCAD score for malignancy discrimination.

	AUC	95% CI	Sensitivity (%)	Specificity (%)	Associated Criterion	Youden Index	*p* Value
iCAD	0.744	0.654 to 0.821	70.1	70.3	>51%	0.404	<0.001

## Data Availability

The data presented in this study are available on request from the corresponding author due to privacy restrictions.

## Abbreviations

The following abbreviations are used in this manuscript:

CEM	Contrast-Enhanced Mammography
MRI	Magnetic Resonance Imaging
BC	Breast Cancer
BI-RADS	Breast Imaging Reporting and Data System
DCIS	Ductal Carcinoma In Situ
NST	No special type (invasive carcinoma of no special type)
VAB	Vacuum-Assisted Biopsy
BPE	Background Parenchymal Enhancement
NME	Non-Mass Enhancement
CC	Craniocaudal
MLO	Mediolateral Oblique
ER	Estrogen Receptor
PR	Progesterone Receptor
HER2	Human Epidermal Growth Factor Receptor 2
LD	Low dose
